# Parallel electrophysiological and prefrontal hemodynamic alterations during stroop task performance in major depressive disorder: a simultaneous EEG-fNIRS study

**DOI:** 10.3389/fpsyt.2026.1884169

**Published:** 2026-08-11

**Authors:** Yang Li, Yan Zhang, Yu Jiang, Gang Cheng, Rong Deng, Lv Yue

**Affiliations:** 1 Security Department, Chengdu Aeronautic Polytechnic University, Chengdu, Sichuan, China; 2 School of Language, Culture, and International Communication, Sichuan Tourism University, Chengdu, Sichuan, China; 3 Office of Student Affairs, Sichuan Nursing Vocational College, Chengdu, Sichuan, China; 4 School of Education, Hunan University of Humanities, Science and Technology, Loudi, Hunan, China; 5 The Fourth People’s Hospital of Chengdu, Chengdu, Sichuan, China; 6 Department of Basic Sciences, Shanxi Medical University, Fenyang, Shanxi, China

**Keywords:** EEG, event-related potentials, executive control, fNIRS, major depressive disorder, prefrontal hemodynamics, stroop task

## Abstract

**Introduction:**

Major depressive disorder (MDD) is associated with executive-control deficits, yet the simultaneous electrophysiological and hemodynamic correlates of Stroop-task performance remain incompletely characterized. This study investigated parallel neural and prefrontal hemodynamic alterations during cognitive conflict processing using simultaneous electroencephalography (EEG) and functional near-infrared spectroscopy (fNIRS).

**Methods:**

Twenty-two patients with MDD and twenty-one healthy controls completed a color-word Stroop task while EEG and fNIRS were recorded simultaneously. Behavioral performance, stimulus-locked event-related potential (ERP) components, oscillatory activity, and prefrontal region-of-interest (ROI) hemoglobin responses were analyzed using group × condition mixed-design analysis of variance.

**Results:**

Compared with healthy controls, patients with MDD showed impaired behavioral performance together with altered ERP components, oscillatory activity, and prefrontal hemoglobin responses during Stroop-task performance, indicating concurrent electrophysiological and hemodynamic abnormalities associated with executive-control dysfunction.

**Discussion:**

These findings support the presence of parallel task-related electrophysiological and prefrontal hemodynamic alterations in MDD during cognitive conflict processing. Larger prospective studies incorporating trial-level reliability metrics, channel-wise fNIRS analyses, and clinical covariates are warranted to further validate these observations.

## 1 Introduction

Major depressive disorder (MDD) is a highly prevalent and disabling psychiatric disorder characterized not only by persistent affective symptoms, but also by broad disturbances in cognition, including impaired attention, slowed information processing, deficient inhibitory control and reduced cognitive flexibility. These cognitive abnormalities are clinically important because they contribute to functional disability, poor psychosocial recovery and incomplete remission even when mood symptoms improve (1–4). Among different cognitive domains, executive control is particularly relevant to depression because it supports the ability to suppress irrelevant information, resolve conflict, monitor errors and flexibly allocate attentional resources. Previous neurocognitive studies have suggested that depressive symptoms are associated with dysfunction of frontocingulate and prefrontal control systems, especially during situations requiring conflict monitoring and response inhibition (1, 3). Therefore, characterizing the neural mechanisms of executive-control impairment may provide insight into the pathophysiology of depression and may help identify candidate physiological measures associated with cognitive dysfunction and symptom burden.

The color-word Stroop task is one of the most widely used paradigms for probing cognitive conflict and inhibitory control. In this task, participants are required to identify the ink color of a word while suppressing the automatic tendency to read its semantic meaning. The incongruent condition, in which word meaning and ink color conflict, places greater demands on selective attention, interference suppression and response monitoring than neutral conditions (5–8). Neuroimaging and electrophysiological studies have shown that Stroop interference engages a distributed cognitive-control network involving the prefrontal cortex, anterior cingulate cortex and related frontoparietal regions (9–11).

Electroencephalography (EEG) provides a temporally precise method for examining the neural dynamics of cognitive control. Because EEG captures neural activity at millisecond resolution, it is particularly suitable for identifying the sequential processing stages involved in Stroop-task performance (12, 13). Event-related potentials (ERPs) have been extensively used to characterize conflict monitoring, attentional allocation and error processing. The N200 component is commonly interpreted as an electrophysiological marker of conflict detection and inhibitory control, especially over frontal and frontocentral regions (14, 15). The P300 component reflects stimulus evaluation, attentional resource allocation and context updating, and is typically maximal over centroparietal regions (16). Error-related negativity (ERN), a response-locked component generated shortly after erroneous responses, is associated with performance monitoring and error detection (17). Previous ERP studies have shown that depression may be associated with reduced P300 amplitude, altered N200 responses and abnormal error-related processing, suggesting that multiple temporal stages of executive control are disrupted in MDD (1, 10, 14, 18). In addition to ERPs, time-frequency EEG measures provide complementary information regarding oscillatory mechanisms of cognitive control. Frontal theta activity has been closely linked to conflict monitoring and control recruitment, whereas alpha-band modulation is associated with attentional regulation and inhibition of task-irrelevant processing (18, 19). Thus, EEG can provide a detailed temporal profile of depression-related abnormalities during cognitive conflict.

Functional near-infrared spectroscopy (fNIRS), in contrast, offers a noninvasive method for measuring task-evoked cortical hemodynamic responses. By detecting concentration changes in oxygenated hemoglobin, deoxygenated hemoglobin and total hemoglobin, fNIRS provides an index of cortical oxygenation and blood-volume regulation during cognitive activity (20–22). The technique is particularly suitable for psychiatric and cognitive neuroscience research because it is relatively tolerant to movement, compatible with naturalistic task settings and can be combined with EEG during simultaneous acquisition (20, 21, 23). Previous fNIRS studies have demonstrated that Stroop interference evokes increased prefrontal activation in healthy individuals, reflecting greater recruitment of executive-control resources during high-conflict processing (9, 24, 25). Reduced or inefficient prefrontal hemodynamic responses have also been observed in psychiatric populations during executive tasks, suggesting that abnormal cortical oxygenation may represent a physiological substrate of cognitive impairment (21, 26). In depression, blunted prefrontal hemodynamic activation may reflect insufficient recruitment of neural resources, altered neurovascular regulation or reduced metabolic support for executive processing. However, because fNIRS measures vascular responses rather than electrical neural activity directly, it cannot by itself determine whether hemodynamic abnormalities are accompanied by parallel electrophysiological deficits.

The simultaneous acquisition of EEG and fNIRS provides an opportunity to investigate cognitive dysfunction from both neural and vascular perspectives. EEG captures rapid electrophysiological events such as N200, P300, ERN and oscillatory activity, whereas fNIRS captures slower hemodynamic responses associated with oxygen delivery and local blood-volume changes (20, 21, 23). Recent simultaneous EEG-fNIRS Stroop studies and open EEG-fNIRS Stroop datasets provide methodological context for analyzing parallel electrophysiological and hemodynamic responses during cognitive-control tasks (27, 28). These two modalities are therefore complementary for examining electrophysiological and hemodynamic aspects of cognitive-control dysfunction, although simultaneous acquisition alone does not establish direct neurovascular coupling.

Despite accumulating evidence from EEG, fNIRS and neuroimaging studies, several important gaps remain. First, many previous studies have examined either electrophysiological or hemodynamic responses separately, making it difficult to determine whether neural and vascular abnormalities co-occur during the same cognitive-control process. Second, although Stroop-task impairment has been repeatedly reported in depression, relatively few studies have simultaneously evaluated behavioral performance, ERP components, oscillatory dynamics, and prefrontal hemodynamic responses within the same experimental framework. Third, the clinical relevance of multimodal physiological measures remains insufficiently understood. If EEG–fNIRS indices are associated with depressive symptom severity, they may provide candidate physiological measures of cognitive dysfunction and may help bridge laboratory-based neurophysiological findings with clinical assessment. These gaps highlight the need for a multimodal approach that links behavioral deficits, electrophysiological abnormalities, hemodynamic alterations and clinical symptom burden during cognitive conflict in MDD.

In the present study, we used simultaneous EEG-fNIRS recording during a color-word Stroop task to characterize electrophysiological and prefrontal hemodynamic responses to cognitive-control demands in patients with MDD, building on prior simultaneous EEG-fNIRS Stroop paradigms and public EEG-fNIRS Stroop data resources (27, 28). Participants with MDD and healthy controls completed neutral and incongruent Stroop trials while behavioral responses, EEG signals, and prefrontal fNIRS signals were recorded concurrently. Behavioral indices included reaction time, accuracy, and Stroop interference scores. EEG analyses focused on stimulus-locked N200 and P300 components and theta/alpha oscillatory activity, and fNIRS analyses focused on ROI-level HbO, HbR, and HbT responses.

## 2 Methods

### 2.1 Participants

A total of 43 participants were enrolled, including 22 patients with major depressive disorder and 21 healthy controls. Patients with MDD were recruited from affiliated clinical services and were diagnosed by trained psychiatrists according to DSM-5 criteria. Healthy controls were recruited from the local community and screened to exclude current or past psychiatric and neurological disorders. All participants had normal or corrected-to-normal vision and were able to complete the Stroop task and simultaneous EEG–fNIRS recording. Participants with bipolar disorder, psychotic disorder, substance-use disorder, neurological disease, severe systemic illness, or incomplete EEG–fNIRS recordings were excluded.

All participants completed clinical symptom assessments before the experimental task. Depressive symptom severity was evaluated using the Self-Rating Depression Scale, Patient Health Questionnaire-9, Hamilton Depression Rating Scale, and Beck Depression Inventory-II. Participants were instructed to avoid alcohol, caffeine, and excessive physical exercise before the experiment to reduce potential physiological confounding effects on EEG and fNIRS signals.

The study was conducted in accordance with the Declaration of Helsinki. All participants provided written informed consent before participation. The protocol was reviewed and approved by the Ethics Committee of Lishui Central Hospital (approval no. 2026(I)-071-01; review date: February 10, 2026).

Demographic and clinical characteristics of the participants are summarized in **Table 1**.

**Table 1:** Demographic and clinical characteristics of participants.

Characteristic	MDD (n = 22)	HC (n = 21)	Statistical test	P value
Age, years	26.18 ± 5.78	26.67 ± 5.28	Welch’s t-test	0.775
Sex, female/male	15/7	11/10	Fisher’s exact test	0.358
Education, years	15.77 ± 2.07	15.86 ± 1.74	Welch’s t-test	0.885
Illness duration, months	33.55 ± 22.23	N/A	Descriptive only	N/A
Psychiatric medication status, n (%)	Receiving: 17 (77.3); not receiving: 5 (22.7)	N/A	Descriptive only	N/A
Episode/course status, n (%)	Acute/first acute: 7 (31.8); partial-remission/recent symptomatic: 9 (40.9); chronic/persistent: 6 (27.3)	N/A	Descriptive only	N/A
Recorded psychiatric comorbidity, n (%)	12 (54.5); mainly anxiety-related diagnoses where specified in clinical records	None reported	Descriptive only	N/A
Controlled medical comorbidity, n (%)	2 (9.1); controlled medical conditions documented as non-exclusionary	None reported	Descriptive only	N/A
SDS score	67.18 ± 5.07	44.19 ± 3.14	Welch’s t-test with FDR correction	<0.001
PHQ-9 score	18.59 ± 2.46	3.48 ± 1.63	Welch’s t-test with FDR correction	<0.001
HAMD score	25.09 ± 2.99	5.29 ± 1.52	Welch’s t-test with FDR correction	<0.001
BDI-II score	36.64 ± 6.95	9.05 ± 3.09	Welch’s t-test with FDR correction	<0.001

Values are presented as mean ± SD, n, or n (%). Age and education were compared using Welch’s t-test, and sex distribution was compared using Fisher’s exact test. Clinical symptom scores were compared using Welch’s t-tests with FDR correction across the four scales. Illness duration, medication status, episode/course status, and comorbidity variables were summarized descriptively for the MDD group and were not entered as covariates in the primary inferential models. Medication status was recorded as current psychiatric medication exposure only; standardized medication classes, doses, and treatment duration were not available in a form suitable for reliable covariate modeling. Recorded psychiatric comorbidities were based on clinical documentation; where specified, they were mainly anxiety-related diagnoses. The available records did not provide complete standardized subtype, severity, onset, treatment-duration, or subgroup details for reliable adjusted or stratified analyses; therefore, comorbidity variables were reported descriptively rather than used for *post hoc* subgroup analyses. MDD, major depressive disorder; HC, healthy controls; N/A, not applicable.

### 2.2 Experimental design

Participants performed a color-word Stroop task during simultaneous EEG and fNIRS recording. The task comprised two analyzed conditions: a neutral condition and an incongruent condition. In the neutral condition, participants viewed non-word letter strings consisting of ‘XXXXX’ presented in one of the task response colors. These stimuli contained no color-word semantic content and served as the non-conflict baseline; therefore, the term neutral refers to a non-word baseline rather than a congruent color-word condition. In the incongruent condition, participants viewed color words presented in a conflicting ink color, such that word meaning and ink color differed. Participants responded according to the ink color rather than the word meaning in all trials.

The task included 64 analyzed trials, with 32 neutral trials and 32 incongruent trials. Behavioral and EEG outcomes were summarized within each condition at the participant level, whereas fNIRS outcomes were summarized at the condition/block level because of the slow hemodynamic response and short intertrial interval. No separate congruent condition was included in the analyzed contrast. This design contrasted a non-semantic color-response baseline with a semantic-conflict condition without introducing an additional congruent color-word category.

### 2.3 Simultaneous EEG–fNIRS acquisition

EEG was recorded using a 64-channel EEG system (actiCHamp, Brain Products GmbH, Gilching, Germany) arranged according to the international 10–20 system. Signals were sampled at 1, 000 Hz, referenced online to FCz, and re-referenced offline to the average reference. Electrode impedance was kept below 10 kΩ throughout recording.

fNIRS was recorded using a multichannel fNIRS system (NIRSport2, NIRx Medical Technologies, Berlin, Germany) with wavelengths of 760 and 850 nm and a sampling rate of 7.81 Hz. The montage comprised 8 sources and 7 detectors forming 20 channels over the prefrontal cortex, with a source–detector distance of approximately 3 cm. Optodes were positioned according to the international 10–20 system and covered bilateral dorsolateral prefrontal and inferior frontal regions. EEG event markers, fNIRS data, and behavioral responses were synchronized using E-Prime 3.0 trigger outputs and Lab Streaming Layer.

The EEG–fNIRS montage was arranged to enable simultaneous assessment of electrophysiological and hemodynamic responses during cognitive conflict processing. EEG electrodes were distributed over the scalp according to a standardized montage, whereas fNIRS optodes were positioned over frontal and prefrontal regions involved in cognitive control, including dorsolateral and inferior frontal regions.

During recording, participants were seated comfortably in front of a computer monitor. They were instructed to minimize head movement and blinking during stimulus presentation. Behavioral responses, EEG signals and fNIRS signals were synchronized with task events for subsequent trial-locked analysis.

### 2.4 Behavioral analysis

Behavioral performance was assessed using reaction time and response accuracy. Mean reaction time and accuracy were analyzed separately for the neutral and incongruent conditions.

RT interference was calculated for each participant as the mean reaction time for correct incongruent trials minus the mean reaction time for correct neutral trials (RT_interference = RT_incongruent - RT_neutral). Accuracy cost was calculated as mean neutral accuracy minus mean incongruent accuracy. Congruent trials were not included because the analyzed contrast consisted of neutral non-word trials and incongruent color-word trials. Accordingly, RT interference in this study denotes interference relative to the neutral baseline rather than traditional incongruent-minus-congruent interference.

Group differences between patients with depression and healthy controls were analyzed for each behavioral measure. In addition, correlations were examined between behavioral indices and clinical symptom scores to determine whether impaired task performance was associated with depressive symptom severity.

### 2.5 EEG preprocessing

EEG preprocessing was performed using EEGLAB 2023.1 running in MATLAB R2022b. Continuous data were band-pass filtered from 0.1 to 30 Hz for ERP analyses and notch-filtered at 50 Hz when necessary. Ocular and muscle artifacts were corrected using independent component analysis; artifact components were removed based on scalp topography, time course, and spectral features. Stimulus-locked epochs were extracted from -200 to 800 ms relative to stimulus onset. Baseline correction used the -200 to 0 ms prestimulus interval. Trials with amplitudes exceeding ±100 μV or residual artifacts were excluded, and only trials with valid behavioral responses were retained. The inferential analyses used participant-level condition summaries generated after preprocessing.

### 2.6 Event-related potential analysis

Event-related potentials were calculated by averaging EEG epochs separately for each participant, group, and task condition. Two primary stimulus-locked ERP components were selected as measures of cognitive-control processing: N200 and P300.

N200 was analyzed as an index of conflict detection and response inhibition. Fz and Cz were selected *a priori* because frontocentral N200 activity is commonly used to index conflict monitoring and inhibitory control in Stroop and related executive-control tasks (14, 15, 18). Peak amplitude and latency were extracted within 200–350 ms. P300 was analyzed as an index of attentional resource allocation and stimulus evaluation, with Pz selected as the primary electrode because P300/P3b responses are typically maximal over centroparietal sites (16, 17). Peak amplitude and latency were extracted within 300–450 ms. ERN was not included as a primary ERP outcome because the number of artifact-free error trials was insufficient for reliable participant-level estimation.

For each ERP component, peak amplitude and latency were extracted within predefined time windows. Group differences and task-condition effects were evaluated to determine whether depression was associated with altered conflict monitoring and stimulus evaluation. Because ERP reliability depends on the number of artifact-free trials, signal-to-noise ratio, component amplitude, and preprocessing choices, component-level estimates based on modest trial numbers should be interpreted cautiously (29, 30).

### 2.7 EEG oscillatory power analysis

Time–frequency analysis was performed to examine task-related oscillatory dynamics. Particular emphasis was placed on theta and alpha frequency bands because of their established roles in cognitive control and attentional regulation.

Theta power was calculated in the 4–8 Hz frequency range over a frontal-midline ROI. Fz and FCz theta values were averaged to reduce single-electrode dependence; this ROI was selected because frontal-midline theta is strongly linked to conflict monitoring and cognitive-control recruitment (18, 19). Although FCz served as the online recording reference, the EEG data were re-referenced offline to the average reference before analysis. Alpha power was calculated in the 8–13 Hz frequency range at Pz because posterior alpha modulation is commonly associated with attentional regulation and task engagement (31).

Task-related changes in theta and alpha power were quantified for neutral and incongruent conditions. Group differences were analyzed using the interaction-first mixed-ANOVA framework to determine whether patients with depression showed altered condition-related theta or alpha modulation during Stroop-task performance.

### 2.8 fNIRS preprocessing

fNIRS preprocessing was performed using Homer3 1.80.2 running in MATLAB R2022b. Raw intensity signals were converted to optical density and inspected for signal quality. Channels were excluded if the scalp-coupling index was below 0.75, if the signal-to-noise ratio was below 30 dB, or if signal saturation occurred for more than 5% of the recording. Motion artifacts were corrected using temporal derivative distribution repair. Corrected signals were band-pass filtered between 0.01 and 0.20 Hz to attenuate slow drift and high-frequency physiological noise. Optical density changes were converted to HbO and HbR concentration changes using the modified Beer–Lambert law with differential pathlength factors of 6.0 for 760 nm and 5.2 for 850 nm. HbT was calculated as HbO + HbR. Hemoglobin responses were normalized and expressed in arbitrary units.

Because the intertrial interval was short and the hemodynamic response is slow, fNIRS responses were summarized at the condition/block level rather than interpreted as isolated single-trial hemodynamic responses. Mean HbO, HbR, and HbT changes were calculated for each condition after subtracting the corresponding precondition/pre-block baseline. Regional responses were averaged across channels covering bilateral dorsolateral prefrontal and inferior frontal regions because these prefrontal areas are consistently implicated in Stroop interference and cognitive-control demand (9, 20, 21, 24, 25).

Optical intensity signals were converted into hemoglobin-related signal changes using the modified Beer–Lambert law and then normalized for group-level analysis. HbO was used as the primary indicator of cortical activation because of its relatively high sensitivity to task-evoked hemodynamic responses. HbR and HbT were analyzed as complementary indicators of oxygen extraction and local blood volume changes.

### 2.9 fNIRS hemodynamic response analysis

The primary fNIRS analysis was conducted at the predefined prefrontal ROI level. HbO, HbR, and HbT participant-level summary values were analyzed by condition and group. Channel-wise spatial inference was not included in the primary analysis.

Regional activation was compared between neutral and incongruent conditions and between patients with MDD and healthy controls. Figures show participant-level ROI distributions that correspond to the statistical summaries used for inference.

### 2.10 Clinical correlation analysis

Clinical symptom scores were correlated with selected neurophysiological measures to examine whether task-related abnormalities were associated with depressive symptom severity. Because P300 amplitude reflects attentional allocation and stimulus evaluation, P300 amplitude during incongruent trials was selected as the primary electrophysiological measure for clinical correlation analysis. Pearson or Spearman correlation was used according to data distribution. To reduce the risk of overinterpreting diagnostic group separation as a within-patient severity effect, both pooled and within-MDD correlations were reported. Correlations were treated as exploratory evidence of possible links between cognitive-control-related neural abnormalities and clinical symptom burden.

### 2.11 Statistical analysis

Statistical analyses were performed using Python 3.11.8. Group × condition mixed-design ANOVA was used for behavioral, ERP, oscillatory, and ROI-level fNIRS summary measures, with group as the between-subject factor and condition as the within-subject factor. All primary group × condition analyses used the neutral and incongruent conditions defined in Section 2.2. Simple-effects tests were FDR-corrected using the Benjamini-Hochberg procedure. No formal *a priori* sample-size calculation was conducted. Therefore, we performed a sensitivity analysis to estimate the minimum detectable effect sizes under the current sample size: Cohen’s d = 0.88 for an independent-samples comparison, r = 0.41 for pooled correlation with n = 43, and r = 0.55 for within-MDD correlation with n = 22. All inferential analyses followed a frequentist framework; Bayes factors were not estimated because no Bayesian model was specified *a priori*.

ERP outcomes included N200 amplitude, N200 latency, P300 amplitude, and P300 latency. Oscillatory outcomes included frontal-midline theta ROI, calculated as the mean of Fz and FCz theta power, and Pz alpha power. fNIRS outcomes were predefined prefrontal ROI-level HbO, HbR, and HbT responses.

**Figure 1:**
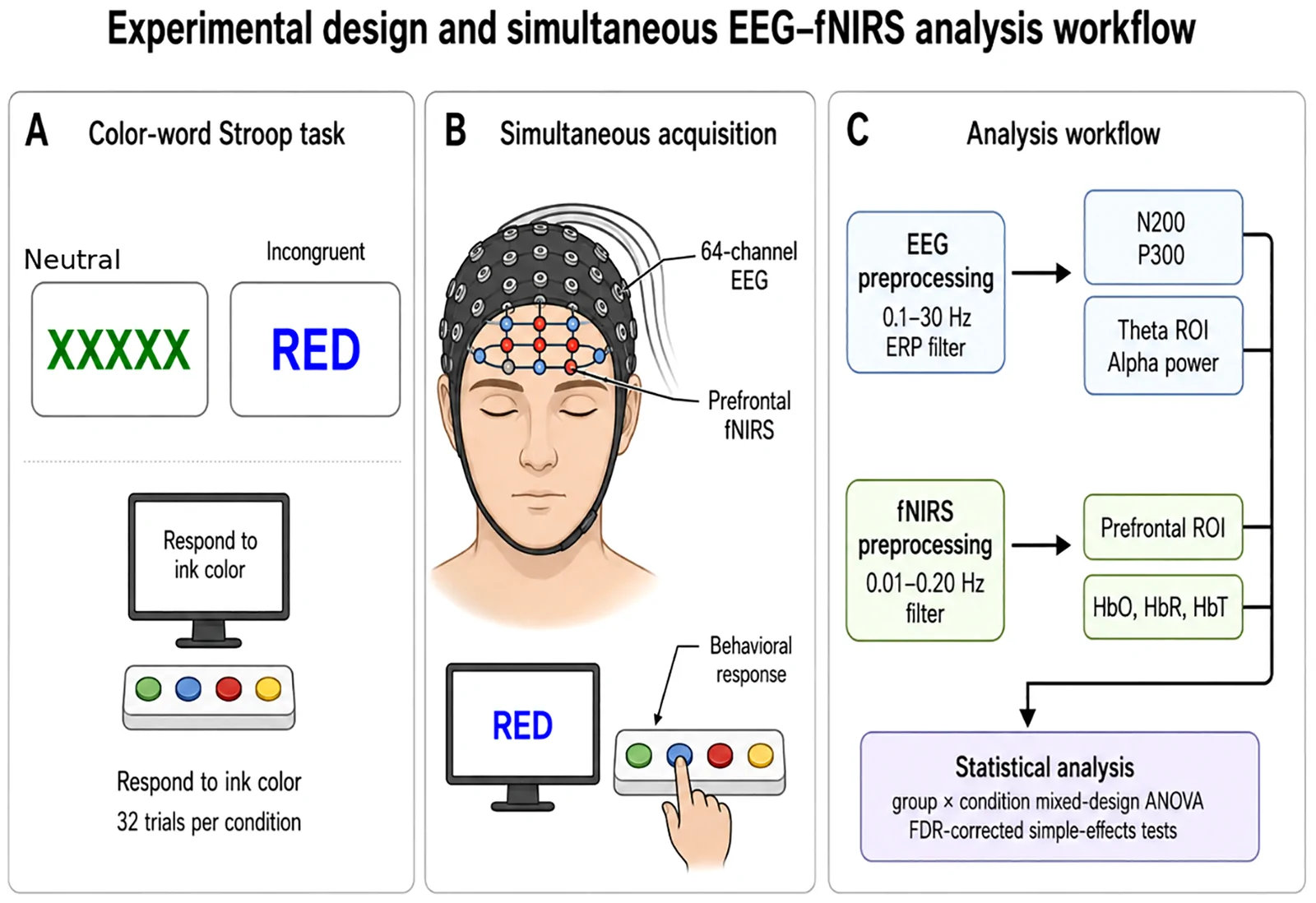
Experimental design and simultaneous EEG–fNIRS analysis workflow. **(A)** Color-word Stroop task paradigm. Participants completed neutral and incongruent trials by responding to the ink color of each stimulus, with 32 trials per condition. **(B)** Simultaneous EEG–fNIRS acquisition. Sixty-four-channel EEG and prefrontal fNIRS signals were recorded simultaneously during Stroop-task performance, together with behavioral responses. **(C)** Data analysis workflow. EEG preprocessing was followed by ERP (N200 and P300) and oscillatory (theta and alpha power) analyses, while fNIRS preprocessing was followed by prefrontal ROI-based HbO, HbR, and HbT analyses. Statistical analyses were performed using group × condition mixed-design ANOVA with FDR-corrected simple-effects tests.

Associations between HAMD and P300 amplitude during incongruent trials were examined using pooled and within-MDD Pearson correlations with 95% confidence intervals. Adjusted regression was not performed because the modest sample size limited reliable multivariable modeling after accounting for the demographic and clinical descriptors summarized in **Table 1**. The overall experimental workflow is illustrated in **Figure 1**.

## 3 Results

### 3.1 Clinical symptom severity

**Figure 2:**
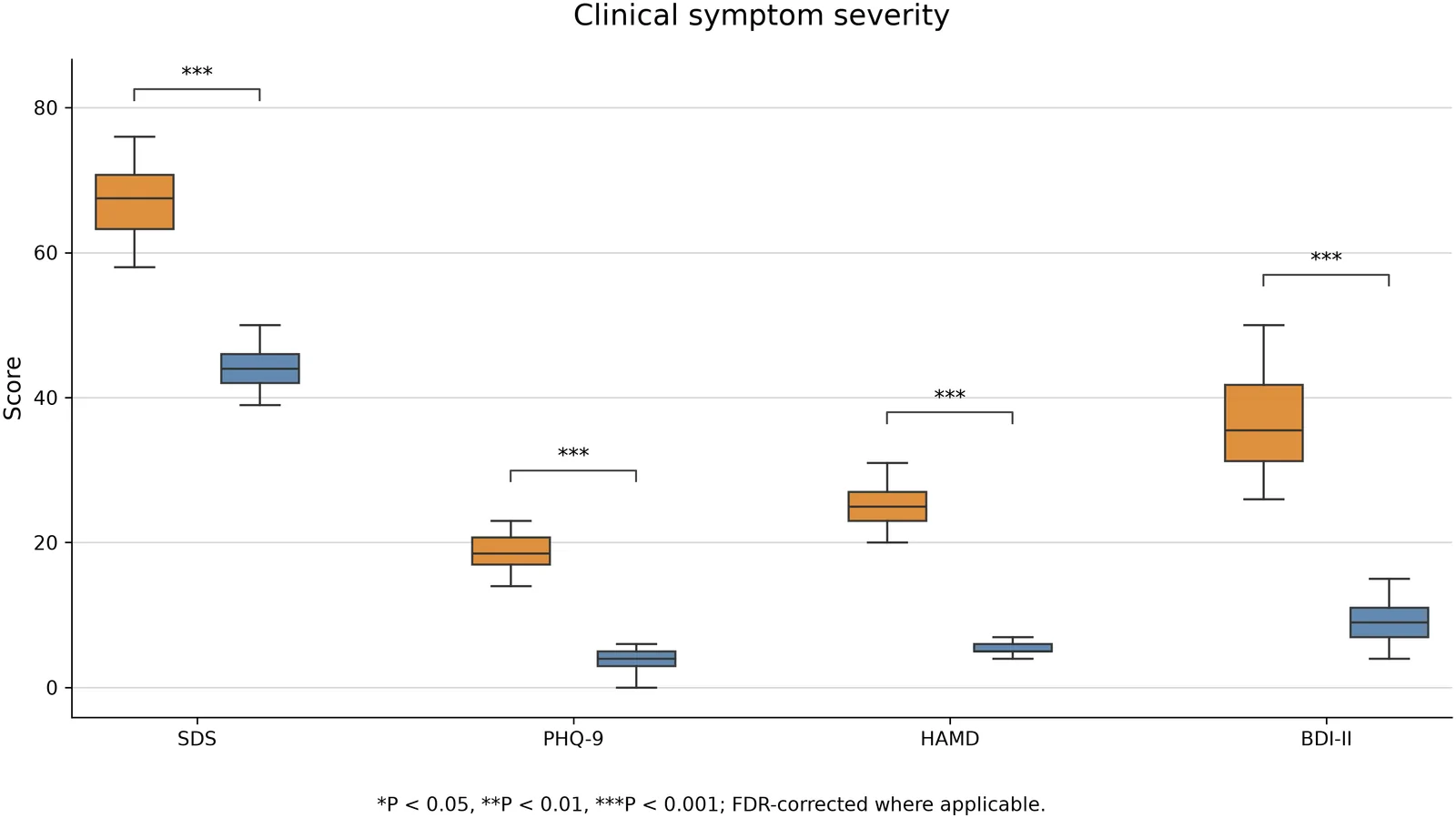
Clinical symptom severity in MDD and HC participants. Boxplots show SDS, PHQ-9, HAMD, and BDI-II scores in patients with major depressive disorder and healthy controls. Between-group comparisons used two-sided Welch’s t-tests with FDR correction across the four clinical scales. Asterisks indicate corrected significance levels: *P < 0.05, **P < 0.01, ***P < 0.001.

Available demographic and clinical characteristics are summarized in **Table 1**. Clinical symptom severity is shown in **Figure 2**. The MDD and HC groups were comparable in age, sex distribution, and education years. In the MDD group, illness duration was 33.55 ± 22.23 months; 17 of 22 participants were receiving psychiatric medication, and 5 were not receiving psychiatric medication at the time of enrollment. Episode/course status and comorbidity information are summarized descriptively in **Table 1** and were not used as covariates in the primary inferential models. Recorded psychiatric comorbidity was present in 12 patients, with clinical documentation indicating mainly anxiety-related diagnoses where specified; controlled medical comorbidity was documented in two patients and was non-exclusionary. Because standardized comorbidity subtype, severity, treatment-duration, and subgroup information was not available for reliable adjusted modeling, the inferential analyses should be interpreted as group-level comparisons rather than medication- or comorbidity-adjusted effects. Clinical scale scores confirmed the expected symptom separation between the MDD and HC groups. Patients with MDD had markedly elevated scores across all four depression-related measures. SDS scores were significantly higher in the MDD group than in HC participants (67.18 ± 5.07 vs. 44.19 ± 3.14; P < 0.001). The same pattern was observed for PHQ-9 (18.59 ± 2.46 vs. 3.48 ± 1.63; P < 0.001), HAMD (25.09 ± 2.99 vs. 5.29 ± 1.52; P < 0.001), and BDI-II scores (36.64 ± 6.95 vs. 9.05 ± 3.09; P < 0.001). These results indicate that the patient group represented a clinically symptomatic depression cohort and provide the expected clinical separation between patients with MDD and healthy controls.

### 3.2 Behavioral performance during the stroop task

**Figure 3:**
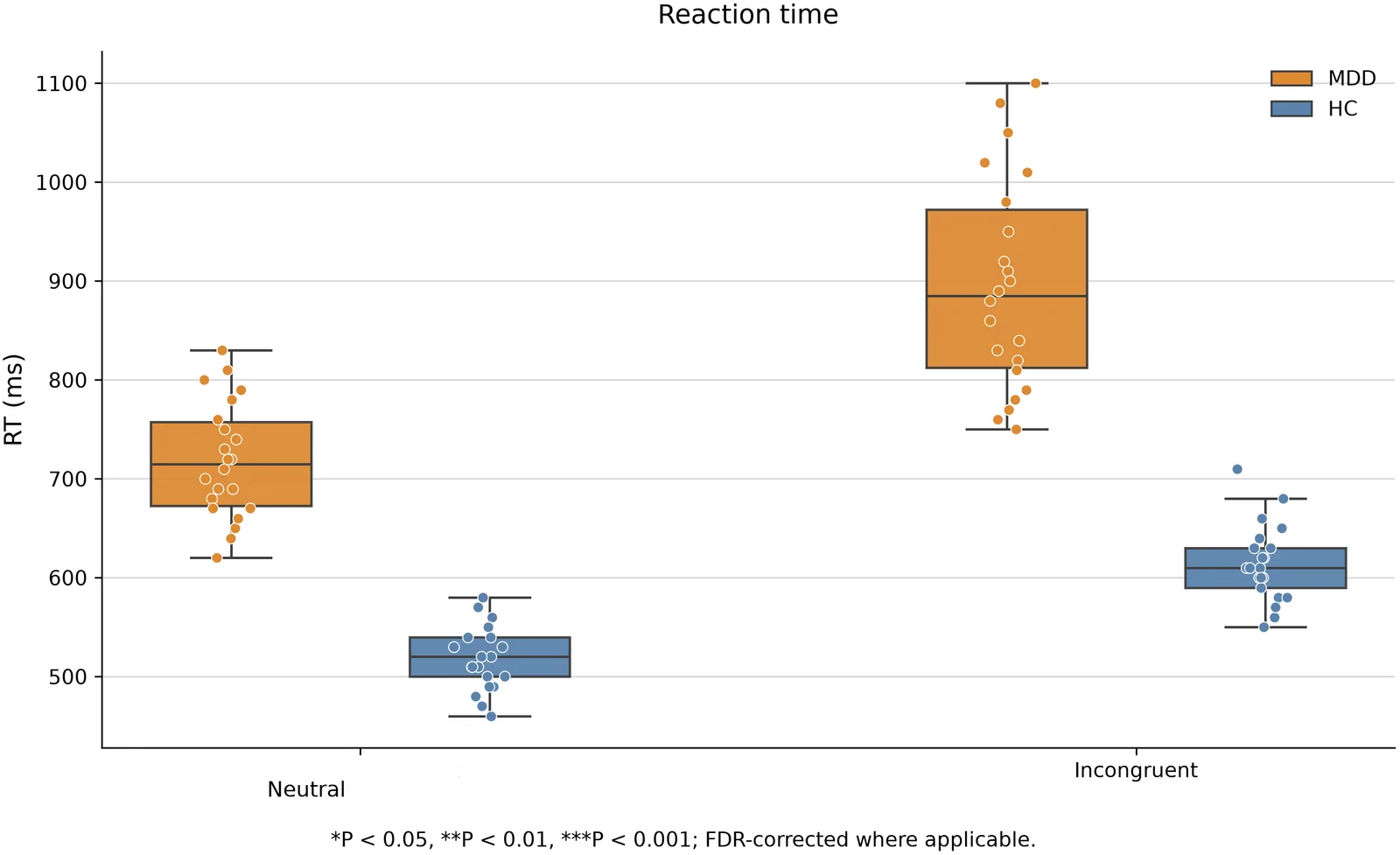
Reaction time during neutral and incongruent Stroop trials. Reaction times were analyzed using group × condition mixed-design ANOVA followed by FDR-corrected simple-effects tests where appropriate. Individual dots represent participants. Boxes indicate median and interquartile range. Asterisks indicate corrected significance levels: *P < 0.05, **P < 0.01, ***P < 0.001.

**Figure 4:**
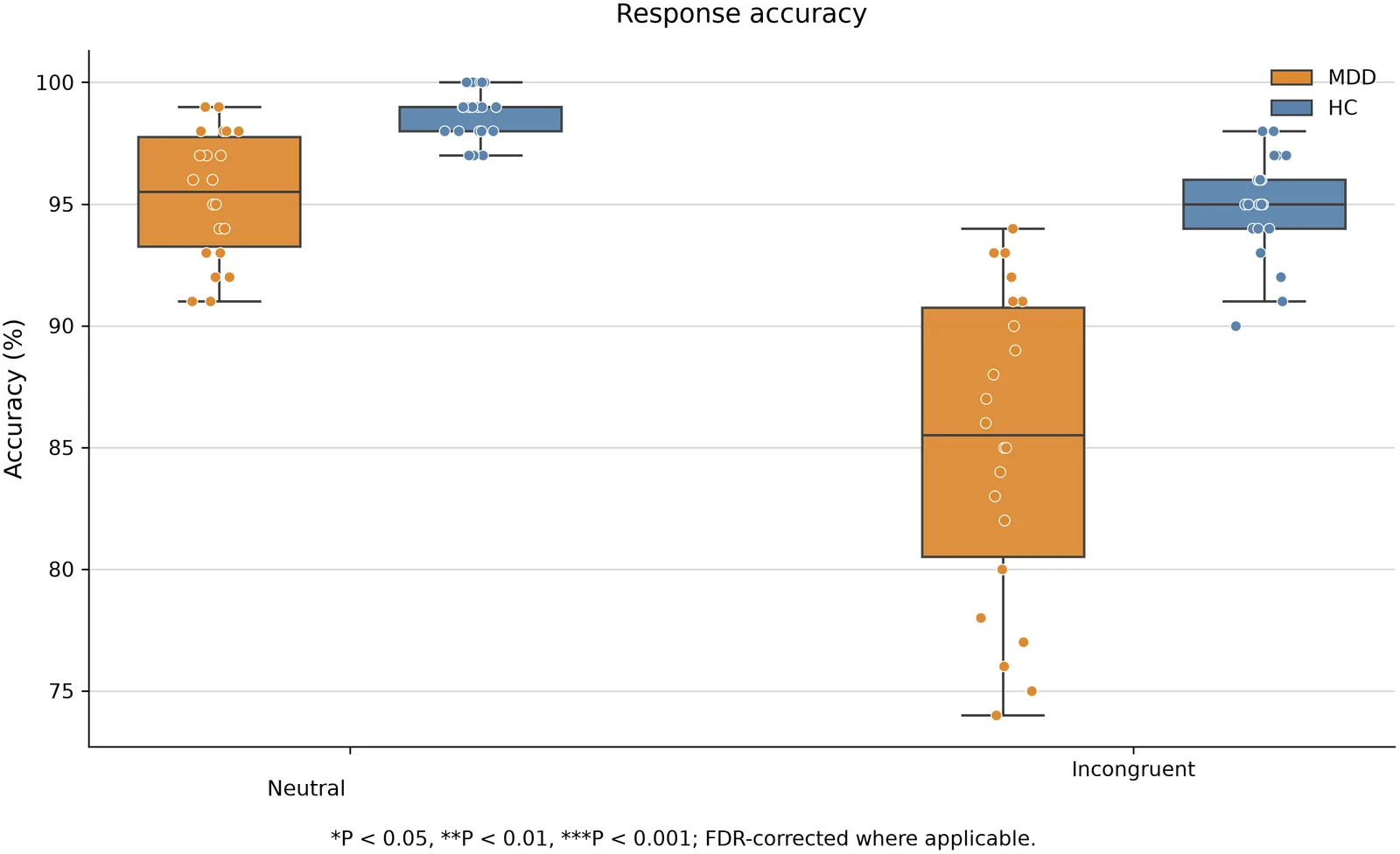
Response accuracy during neutral and incongruent Stroop trials. Accuracy was analyzed using group × condition mixed-design ANOVA followed by FDR-corrected simple-effects tests where appropriate. Individual dots represent participants. Boxes indicate median and interquartile range. Asterisks indicate corrected significance levels: *P < 0.05, **P < 0.01, ***P < 0.001.

Reaction time showed a group × condition interaction, F(1, 41) = 44.14, P < 0.001, partial eta squared = 0.518; a group main effect, F(1, 41) = 153.49, P < 0.001, partial eta squared = 0.789; and a condition main effect, F(1, 41) = 490.56, P < 0.001, partial eta squared = 0.923. Accuracy showed a group × condition interaction, F(1, 41) = 52.76, P < 0.001, partial eta squared = 0.563; a group main effect, F(1, 41) = 41.56, P < 0.001, partial eta squared = 0.503; and a condition main effect, F(1, 41) = 247.18, P < 0.001, partial eta squared = 0.858. RT interference was defined as mean correct incongruent RT minus mean correct neutral RT, and accuracy cost was defined as mean neutral accuracy minus mean incongruent accuracy. Because no congruent condition was included, RT interference represents incongruent-minus-neutral interference in the present task. Behavioral performance is shown in **Figures 3**, **4**. Full behavioral ANOVA, FDR-corrected simple-effects, and descriptive results are provided in **Supplementary Table 2a–S2c**.

### 3.3 ERP signatures of conflict monitoring and stimulus evaluation

**Figure 5:**
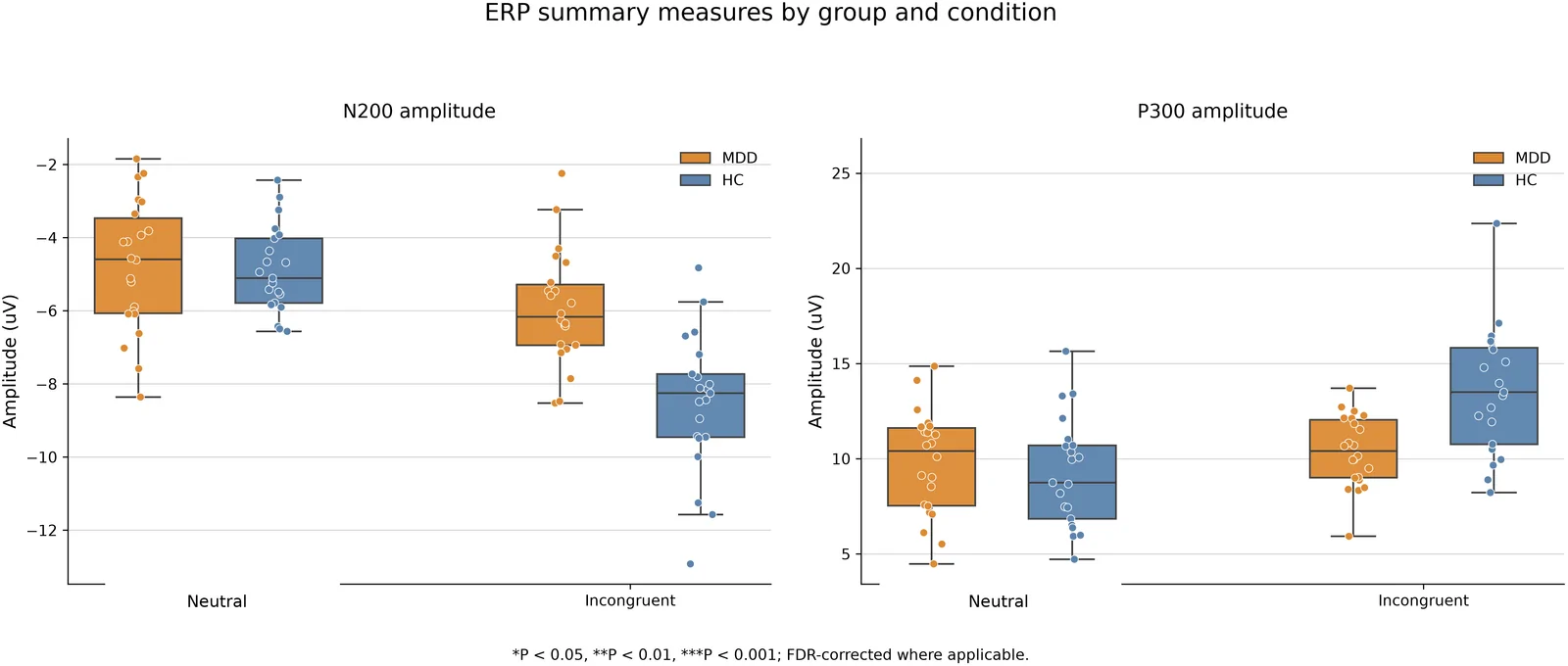
ERP summary measures during Stroop-task processing. N200 and P300 amplitudes are shown by group and condition. Individual dots represent participants. Boxes indicate median and interquartile range. Statistical analyses were performed using group × condition mixed-design ANOVA followed by FDR-corrected simple-effects tests where appropriate. Asterisks indicate corrected significance levels: *P < 0.05, **P < 0.01, ***P < 0.001.

ERP summary analyses showed the following mixed-design ANOVA results. ERP results are shown in **Figure 5**. N200 amplitude: group × condition interaction: F(1, 41) = 11.75, P = 0.001, partial eta squared = 0.223; group main effect: F(1, 41) = 15.18, P < 0.001, partial eta squared = 0.270; condition main effect: F(1, 41) = 44.11, P < 0.001, partial eta squared = 0.518. N200 latency: group × condition interaction: F(1, 41) = 0.08, P = 0.785, partial eta squared = 0.002; group main effect: F(1, 41) = 0.83, P = 0.367, partial eta squared = 0.020; condition main effect: F(1, 41) = 1.17, P = 0.286, partial eta squared = 0.028. P300 amplitude: group × condition interaction: F(1, 41) = 13.59, P < 0.001, partial eta squared = 0.249; group main effect: F(1, 41) = 4.53, P = 0.039, partial eta squared = 0.099; condition main effect: F(1, 41) = 22.30, P < 0.001, partial eta squared = 0.352. P300 latency: group × condition interaction: F(1, 41) = 2.95, P = 0.093, partial eta squared = 0.067; group main effect: F(1, 41) = 36.21, P < 0.001, partial eta squared = 0.469; condition main effect: F(1, 41) = 1.09, P = 0.302, partial eta squared = 0.026. Because ERP outcomes were defined as predefined component summaries, these results should be interpreted as component-level analyses rather than electrode-factor analyses. Full ERP ANOVA, FDR-corrected simple-effects, and descriptive results are provided in **Supplementary Tables 3**, **S3b, S4a**, and **S4b**.

ERN was not interpreted as a primary outcome because the number of artifact-free error trials was insufficient for reliable participant-level estimation in most participants. Accordingly, response-locked error-related activity was not used to support the main conclusions.

Condition-specific visual summaries of ERP amplitudes, ERP latencies, and prefrontal hemodynamic responses during incongruent trials are provided in **Supplementary Figures 1-3**.

### 3.4 Task-related oscillatory dynamics

**Figure 6:**
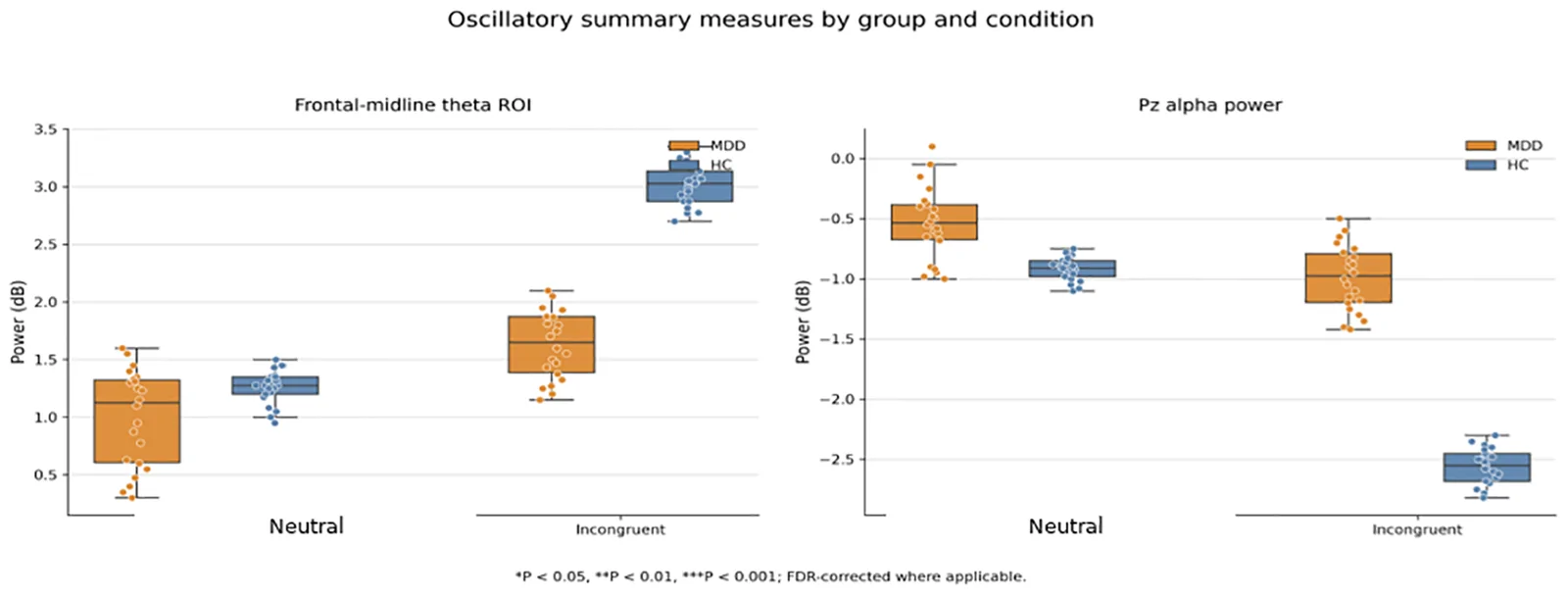
Oscillatory power during Stroop-task processing. The main panels show frontal-midline theta ROI and Pz alpha power by group and condition. Individual dots represent participants. Boxes indicate median and interquartile range. Statistical analyses were performed using group × condition mixed-design ANOVA followed by FDR-corrected simple-effects tests where appropriate. Asterisks indicate corrected significance levels: *P < 0.05, **P < 0.01, ***P < 0.001.

Significant group × condition interactions were observed for frontal-midline theta ROI and Pz alpha power. Oscillatory results are shown in **Figure 6**. Full oscillatory ANOVA, FDR-corrected simple-effects, and descriptive results are provided in **Supplementary Tables 5a–S5c**. These summary results support task-related oscillatory alterations; conflict-related interpretation should be based on the group × condition interaction rather than isolated between-group comparisons within one condition.

### 3.5 Prefrontal hemodynamic responses

**Figure 7:**
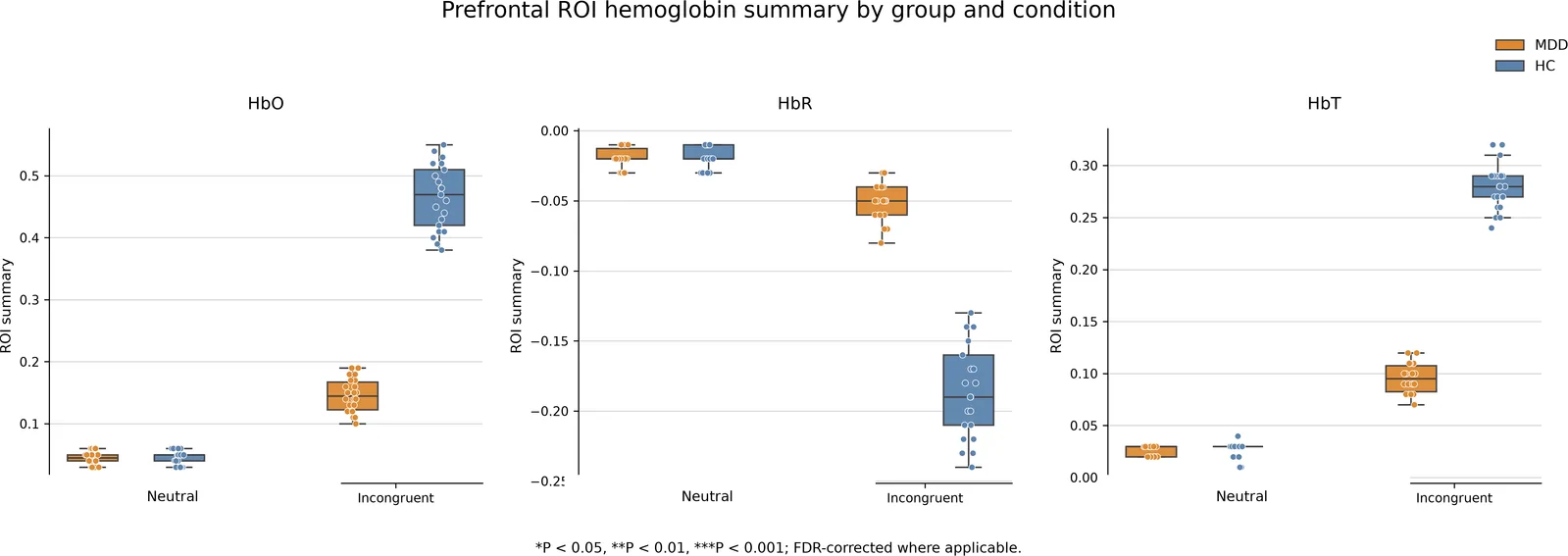
Prefrontal ROI hemoglobin summary during Stroop-task processing. HbO, HbR, and HbT are shown by group and condition. Individual dots represent participants. Boxes indicate median and interquartile range. Statistical analyses were performed using group × condition mixed-design ANOVA followed by FDR-corrected simple-effects tests where appropriate. Asterisks indicate corrected significance levels: *P < 0.05, **P < 0.01, ***P < 0.001.

Significant group × condition interactions were observed for HbO, HbR, and HbT. fNIRS results are shown in **Figure 7**. Full prefrontal ROI fNIRS ANOVA, FDR-corrected simple-effects, and descriptive results are provided in **Supplementary Tables 6a–S6c**. These results support prefrontal ROI-level hemodynamic alterations during Stroop-task processing.

### 3.6 Association between electrophysiological impairment and symptom severity

**Figure 8:**
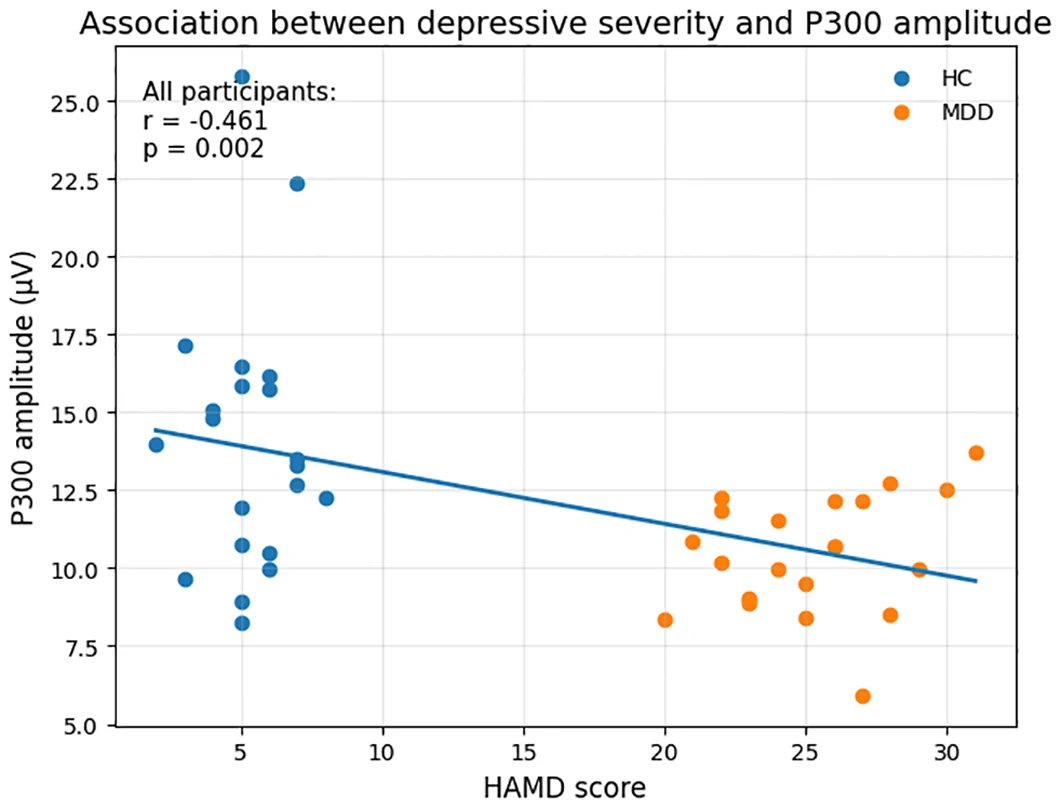
Association between depressive severity and P300 amplitude. Scatterplot showing the relationship between HAMD score and P300 amplitude during incongruent trials in healthy controls (HC) and patients with major depressive disorder (MDD). Blue dots indicate HC participants and orange dots indicate MDD participants. The solid regression line represents the overall linear relationship across all participants. An exploratory pooled Pearson correlation showed a negative association between HAMD score and P300 amplitude (r = -0.461, P = 0.002). Because HAMD scores differed markedly between diagnostic groups, this association should be interpreted cautiously and requires confirmation within the MDD group and in models including clinical covariates.

P300 amplitude during incongruent trials was correlated with HAMD scores. The pooled correlation across all participants was r = -0.461 (95% CI -0.669 to -0.186; P = 0.002; FDR-corrected P = 0.004). Within the MDD group, the correlation was r = 0.289 (95% CI -0.151 to 0.633; P = 0.192; FDR-corrected P = 0.192). Correlation analysis is shown in **Figure 8**. Adjusted regression was not performed because the modest sample size limited reliable multivariable modeling after accounting for the demographic and clinical descriptors summarized in **Table 1**. The pooled association may partly reflect diagnostic group separation rather than a within-MDD symptom-severity relationship. Clinical correlation and sensitivity-analysis results are summarized in **Supplementary Table 7**.

## 4 Discussion

This study used simultaneous EEG-fNIRS recording during a Stroop task to examine cognitive-control abnormalities in patients with MDD. Using participant-level condition summaries, we found that patients with MDD showed slower and less accurate task performance, altered N200 and P300 responses, altered frontal-midline theta and alpha power, and altered prefrontal ROI hemoglobin responses. Findings were interpreted as conflict-related when supported by significant group × condition interactions and otherwise as task-related alterations.

The behavioral and ERP findings are consistent with previous studies showing impaired conflict processing and frontocingulate dysfunction in depression. Holmes and Pizzagalli reported abnormal response-conflict processing in unmedicated patients with major depression, suggesting that depression is associated with dysfunction of frontal cognitive-control systems (1). Broader reviews and meta-analyses have also indicated that executive dysfunction, attentional impairment and slowed information processing are common features of MDD (2–4). In the context of Stroop-task processing, previous ERP studies have shown that N200 is closely related to conflict detection and inhibitory control, whereas P300 reflects attentional allocation and stimulus evaluation (10, 14–16). In line with these studies, the present work found reduced N200 and P300 responses in MDD, indicating weakened conflict monitoring and impaired allocation of cognitive resources. Unlike studies using only behavioral or ERP measures, the present study showed that electrophysiological alterations occurred alongside reduced task accuracy and prolonged reaction time. However, because brain–behavior correlations were not established for all outcomes, the findings should be interpreted as parallel behavioral and neurophysiological abnormalities rather than direct evidence that specific neural deficits caused task impairment.

The fNIRS results also agree with earlier neuroimaging studies showing abnormal prefrontal activation during executive-control tasks in depression and are broadly consistent with previous simultaneous EEG-fNIRS and fNIRS investigations (32–39). Schroeter and colleagues demonstrated that fNIRS can detect prefrontal activation during color-word Stroop interference and that prefrontal oxygenation increases with cognitive-control demand (9, 25). Ehlis et al. further emphasized the value of fNIRS for detecting prefrontal dysfunction in psychiatric disorders (21). Consistent with these findings, the present study showed that HC participants exhibited stronger HbO and HbT responses during incongruent trials, whereas MDD participants showed a blunted hemodynamic response. These results should be interpreted as ROI-level prefrontal hemodynamic alterations observed alongside electrophysiological differences, rather than as evidence of direct cross-modal coupling.

A strength of this study is the simultaneous acquisition of EEG and fNIRS during the same cognitive-control task, which allowed electrophysiological and prefrontal hemodynamic responses to be examined within a unified experimental framework. Nevertheless, the present analysis was primarily activation-based and did not quantify trial-by-trial EEG-fNIRS coupling. Because direct EEG-fNIRS coupling was not estimated, the present findings should be interpreted as parallel electrophysiological and prefrontal hemodynamic alterations rather than direct evidence of altered neurovascular coupling.

Several limitations should be considered. First, the sample size was modest and no formal *a priori* sample-size calculation was available, although sensitivity analysis was added for the current sample. Second, the present analyses were based on participant-level condition summaries, which limited additional trial-level reliability analyses and channel-wise fNIRS spatial inference. Third, available demographic and clinical descriptors were summarized in **Table 1**, but medication status, episode/course status, comorbidity, sleep variables, and acquisition time were not incorporated as covariates in the primary inferential models; sleep variables and acquisition time were not systematically recorded. Therefore, the findings should be interpreted as group-level associations observed in this clinical cohort rather than medication-naive, comorbidity-adjusted, or sleep-adjusted MDD-specific effects. Future studies integrating EEG-fNIRS connectivity analyses, including functional and effective connectivity approaches, may further clarify neurovascular interactions during cognitive control (33, 34, 40–44).

## 5 Conclusion

In conclusion, the behavioral, EEG, and fNIRS summaries indicate that patients with MDD show impaired Stroop-task performance together with task-related electrophysiological and prefrontal hemodynamic alterations in a neutral-versus-incongruent Stroop contrast. The pooled association between lower P300 amplitude and higher HAMD scores should be interpreted cautiously because it may partly reflect diagnostic group separation rather than a within-MDD symptom-severity relationship. Larger prospective studies with trial-level reliability metrics, channel-wise fNIRS spatial analysis, direct EEG-fNIRS coupling models, and clinical covariate adjustment are needed before stronger mechanistic, biomarker-level, neurovascular-coupling, or clinical-application claims can be made.

## Data Availability

The datasets presented in this article are not readily available because they contain sensitive clinical and neurophysiological information from patients with major depressive disorder, including psychiatric symptom scores, behavioral performance, and synchronized EEG–fNIRS recordings. De-identified data may be made available from the corresponding author upon reasonable request, subject to institutional ethical approval and appropriate data-use agreements.
