# Supplementary material for: Parallel electrophysiological and prefrontal hemodynamic alterations during stroop task performance in major depressive disorder: a simultaneous EEG-fNIRS study
Source: Front Psychiatry. 2026 Aug 11;17:1884169. doi: 10.3389/fpsyt.2026.1884169 (PMC13504079; doi:10.3389/fpsyt.2026.1884169)
Supplement: Supplementary file 1 [file Supplementaryfile1.docx]

Supplementary Information

Manuscript title:

Parallel Electrophysiological and Prefrontal Hemodynamic Alterations During Stroop Task Performance in Major Depressive Disorder: A Simultaneous EEG-fNIRS Study

Authors:

Li Yang, Jiang Yu, Zhang Yan, Cheng Gang, Deng Rong, Yue Lv

Correspondence:

Li Yang: liyang2180008@163.com

Yue Lv: lvy960630@163.com

# Section 1. Supplementary Figures

## Supplementary Figure 1. ERP amplitude differences between MDD and HC participants during incongruent trials.

This figure is retained only as a condition-specific visual summary; primary inference is based on the group × condition analyses reported in Figure 5 and the Results. Asterisks indicate corrected significance levels where shown.

## Supplementary Figure 2. ERP latency differences between MDD and HC participants during incongruent trials.

This figure is retained only as a condition-specific visual summary; primary inference is based on the group × condition analyses and supplementary latency summary. Asterisks indicate corrected significance levels where shown.

## Supplementary Figure 3. Condition-specific prefrontal hemodynamic summary during incongruent trials.

This figure is retained only as a condition-specific visual summary; primary inference is based on the group × condition ROI analyses reported in Figure 7 and the Results. Asterisks indicate corrected significance levels where shown.

# Section 2. Supplementary Tables

Unless otherwise specified, the neutral condition in the supplementary tables refers to the colored 'XXXXX' non-word baseline described in the main manuscript; no separate congruent condition was included in the analyzed contrast.

## Supplementary Table S1. Clinical symptom severity comparisons.

| Measure | MDD, mean ± SD | HC, mean ± SD | Statistical test | P value | FDR-corrected P value | Effect size |
| --- | --- | --- | --- | --- | --- | --- |
| SDS | 67.18 ± 5.07 | 44.19 ± 3.14 | Welch's t-test with FDR correction across the four clinical scales | <0.001 | <0.001 | Not calculated |
| PHQ-9 | 18.59 ± 2.46 | 3.48 ± 1.63 | Welch's t-test with FDR correction across the four clinical scales | <0.001 | <0.001 | Not calculated |
| HAMD | 25.09 ± 2.99 | 5.29 ± 1.52 | Welch's t-test with FDR correction across the four clinical scales | <0.001 | <0.001 | Not calculated |
| BDI-II | 36.64 ± 6.95 | 9.05 ± 3.09 | Welch's t-test with FDR correction across the four clinical scales | <0.001 | <0.001 | Not calculated |

Note. Data are participant-level condition-summary measures unless otherwise specified. Mixed-design ANOVA used group as the between-subject factor and condition as the within-subject factor. Simple-effects P values were FDR-corrected using the Benjamini-Hochberg procedure where applicable. MDD = major depressive disorder; HC = healthy controls; ROI = region of interest. Effect sizes for the clinical-scale separation checks were not calculated because these comparisons were included to document clinical group separation rather than as primary neurophysiological outcomes.

## Supplementary Table S2a. Behavioral mixed-design ANOVA results.

| Outcome | Effect | F | df1 | df2 | P value | FDR-corrected P value | Partial eta squared |
| --- | --- | --- | --- | --- | --- | --- | --- |
| Reaction time | Group | 153.488 | 1 | 41 | <0.001 | <0.001 | 0.789 |
| Reaction time | Condition | 490.562 | 1 | 41 | <0.001 | <0.001 | 0.923 |
| Reaction time | Interaction | 44.137 | 1 | 41 | <0.001 | <0.001 | 0.518 |
| Accuracy | Group | 41.556 | 1 | 41 | <0.001 | <0.001 | 0.503 |
| Accuracy | Condition | 247.181 | 1 | 41 | <0.001 | <0.001 | 0.858 |
| Accuracy | Interaction | 52.763 | 1 | 41 | <0.001 | <0.001 | 0.563 |

Note. Data are participant-level condition-summary measures unless otherwise specified. Mixed-design ANOVA used group as the between-subject factor and condition as the within-subject factor. Simple-effects P values were FDR-corrected using the Benjamini-Hochberg procedure where applicable. MDD = major depressive disorder; HC = healthy controls; ROI = region of interest.

## Supplementary Table S2b. Behavioral FDR-corrected simple-effects results.

| Outcome | Comparison | n1 | n2 | t | P value | FDR-corrected P value | Cohen's d |
| --- | --- | --- | --- | --- | --- | --- | --- |
| Reaction time | MDD vs HC within neutral | 22 | 21 | 13.881 | <0.001 | <0.001 | 4.182 |
| Reaction time | MDD vs HC within incongruent | 22 | 21 | 11.530 | <0.001 | <0.001 | 3.454 |
| Reaction time | incongruent vs neutral within MDD | 22 | 22 | 15.429 | <0.001 | <0.001 | 3.289 |
| Reaction time | incongruent vs neutral within HC | 21 | 21 | 24.044 | <0.001 | <0.001 | 5.247 |
| Accuracy | MDD vs HC within neutral | 22 | 21 | -5.581 | <0.001 | <0.001 | -1.673 |
| Accuracy | MDD vs HC within incongruent | 22 | 21 | -6.887 | <0.001 | <0.001 | -2.062 |
| Accuracy | incongruent vs neutral within MDD | 22 | 22 | -12.330 | <0.001 | <0.001 | -2.629 |
| Accuracy | incongruent vs neutral within HC | 21 | 21 | -12.637 | <0.001 | <0.001 | -2.758 |

Note. Data are participant-level condition-summary measures unless otherwise specified. Mixed-design ANOVA used group as the between-subject factor and condition as the within-subject factor. Simple-effects P values were FDR-corrected using the Benjamini-Hochberg procedure where applicable. MDD = major depressive disorder; HC = healthy controls; ROI = region of interest.

## Supplementary Table S2c. Behavioral descriptive statistics by group and condition.

| Outcome | Group | Condition | n | Mean | SD | SEM |
| --- | --- | --- | --- | --- | --- | --- |
| Reaction time | MDD | neutral | 22 | 718.64 | 58.50 | 12.47 |
| Reaction time | MDD | incongruent | 22 | 895.45 | 107.11 | 22.84 |
| Reaction time | HC | neutral | 21 | 519.52 | 32.48 | 7.09 |
| Reaction time | HC | incongruent | 21 | 614.29 | 39.19 | 8.55 |
| Accuracy | MDD | neutral | 22 | 95.36 | 2.57 | 0.55 |
| Accuracy | MDD | incongruent | 22 | 85.14 | 6.32 | 1.35 |
| Accuracy | HC | neutral | 21 | 98.67 | 1.02 | 0.22 |
| Accuracy | HC | incongruent | 21 | 94.95 | 2.13 | 0.47 |

Note. Descriptive statistics were computed from participant-level condition-summary values.

## Supplementary Table S3. ERP mixed-design ANOVA results.

| Outcome | Effect | F | df1 | df2 | P value | FDR-corrected P value | Partial eta squared |
| --- | --- | --- | --- | --- | --- | --- | --- |
| P300 amplitude | Group | 4.525 | 1 | 41 | 0.039 | 0.043 | 0.099 |
| P300 amplitude | Condition | 22.304 | 1 | 41 | <0.001 | <0.001 | 0.352 |
| P300 amplitude | Interaction | 13.589 | 1 | 41 | <0.001 | <0.001 | 0.249 |
| P300 latency | Group | 36.206 | 1 | 41 | <0.001 | <0.001 | 0.469 |
| P300 latency | Condition | 1.093 | 1 | 41 | 0.302 | 0.302 | 0.026 |
| P300 latency | Interaction | 2.953 | 1 | 41 | 0.093 | 0.103 | 0.067 |
| N200 amplitude | Group | 15.177 | 1 | 41 | <0.001 | <0.001 | 0.270 |
| N200 amplitude | Condition | 44.110 | 1 | 41 | <0.001 | <0.001 | 0.518 |
| N200 amplitude | Interaction | 11.754 | 1 | 41 | 0.001 | 0.002 | 0.223 |
| N200 latency | Group | 0.831 | 1 | 41 | 0.367 | 0.367 | 0.020 |
| N200 latency | Condition | 1.170 | 1 | 41 | 0.286 | 0.302 | 0.028 |
| N200 latency | Interaction | 0.075 | 1 | 41 | 0.785 | 0.785 | 0.002 |

Note. Data are participant-level condition-summary measures unless otherwise specified. Mixed-design ANOVA used group as the between-subject factor and condition as the within-subject factor. Simple-effects P values were FDR-corrected using the Benjamini-Hochberg procedure where applicable. MDD = major depressive disorder; HC = healthy controls; ROI = region of interest.

## Supplementary Table S3b. ERP descriptive statistics by group and condition.

| Outcome | Group | Condition | n | Mean | SD | SEM |
| --- | --- | --- | --- | --- | --- | --- |
| N200 amplitude | MDD | neutral | 22 | -4.76 | 1.79 | 0.38 |
| N200 amplitude | MDD | incongruent | 22 | -5.94 | 1.55 | 0.33 |
| N200 amplitude | HC | neutral | 21 | -4.89 | 1.18 | 0.26 |
| N200 amplitude | HC | incongruent | 21 | -8.52 | 1.91 | 0.42 |
| N200 latency | MDD | neutral | 22 | 286.68 | 20.45 | 4.36 |
| N200 latency | MDD | incongruent | 22 | 290.18 | 22.72 | 4.84 |
| N200 latency | HC | neutral | 21 | 281.76 | 15.51 | 3.39 |
| N200 latency | HC | incongruent | 21 | 287.62 | 18.32 | 4.00 |
| P300 amplitude | MDD | neutral | 22 | 9.77 | 2.76 | 0.59 |
| P300 amplitude | MDD | incongruent | 22 | 10.40 | 1.87 | 0.40 |
| P300 amplitude | HC | neutral | 21 | 9.25 | 2.87 | 0.63 |
| P300 amplitude | HC | incongruent | 21 | 14.05 | 4.26 | 0.93 |
| P300 latency | MDD | neutral | 22 | 336.27 | 35.70 | 7.61 |
| P300 latency | MDD | incongruent | 22 | 350.45 | 22.90 | 4.88 |
| P300 latency | HC | neutral | 21 | 313.33 | 18.86 | 4.12 |
| P300 latency | HC | incongruent | 21 | 309.62 | 13.66 | 2.98 |

Note. Descriptive statistics were computed from participant-level condition-summary values.

## Supplementary Table S4a. ERP amplitude FDR-corrected simple-effects results.

| Outcome | Comparison | n1 | n2 | t | P value | FDR-corrected P value | Cohen's d |
| --- | --- | --- | --- | --- | --- | --- | --- |
| P300 amplitude | MDD vs HC within neutral | 22 | 21 | 0.597 | 0.554 | 0.554 | 0.182 |
| P300 amplitude | MDD vs HC within incongruent | 22 | 21 | -3.609 | 0.001 | 0.002 | -1.119 |
| P300 amplitude | incongruent vs neutral within MDD | 22 | 22 | 0.972 | 0.342 | 0.456 | 0.207 |
| P300 amplitude | incongruent vs neutral within HC | 21 | 21 | 5.138 | <0.001 | <0.001 | 1.121 |
| N200 amplitude | MDD vs HC within neutral | 22 | 21 | 0.265 | 0.793 | 0.793 | 0.080 |
| N200 amplitude | MDD vs HC within incongruent | 22 | 21 | 4.845 | <0.001 | <0.001 | 1.485 |
| N200 amplitude | incongruent vs neutral within MDD | 22 | 22 | -2.603 | 0.017 | 0.022 | -0.555 |
| N200 amplitude | incongruent vs neutral within HC | 21 | 21 | -6.501 | <0.001 | <0.001 | -1.419 |

Note. Data are participant-level condition-summary measures unless otherwise specified. Mixed-design ANOVA used group as the between-subject factor and condition as the within-subject factor. Simple-effects P values were FDR-corrected using the Benjamini-Hochberg procedure where applicable. MDD = major depressive disorder; HC = healthy controls; ROI = region of interest.

## Supplementary Table S4b. ERP latency FDR-corrected simple-effects results.

| Outcome | Comparison | n1 | n2 | t | P value | FDR-corrected P value | Cohen's d |
| --- | --- | --- | --- | --- | --- | --- | --- |
| P300 latency | MDD vs HC within neutral | 22 | 21 | 2.651 | 0.012 | 0.025 | 0.798 |
| P300 latency | MDD vs HC within incongruent | 22 | 21 | 7.139 | <0.001 | <0.001 | 2.153 |
| P300 latency | incongruent vs neutral within MDD | 22 | 22 | 1.621 | 0.120 | 0.160 | 0.346 |
| P300 latency | incongruent vs neutral within HC | 21 | 21 | -0.683 | 0.502 | 0.502 | -0.149 |
| N200 latency | MDD vs HC within neutral | 22 | 21 | 0.891 | 0.378 | 0.685 | 0.270 |
| N200 latency | MDD vs HC within incongruent | 22 | 21 | 0.408 | 0.685 | 0.685 | 0.124 |
| N200 latency | incongruent vs neutral within MDD | 22 | 22 | 0.546 | 0.591 | 0.685 | 0.116 |
| N200 latency | incongruent vs neutral within HC | 21 | 21 | 1.030 | 0.315 | 0.685 | 0.225 |

Note. Data are participant-level condition-summary measures unless otherwise specified. Mixed-design ANOVA used group as the between-subject factor and condition as the within-subject factor. Simple-effects P values were FDR-corrected using the Benjamini-Hochberg procedure where applicable. MDD = major depressive disorder; HC = healthy controls; ROI = region of interest.

## Supplementary Table S5a. Oscillatory mixed-design ANOVA results.

| Outcome | Effect | F | df1 | df2 | P value | FDR-corrected P value | Partial eta squared |
| --- | --- | --- | --- | --- | --- | --- | --- |
| Frontal-midline theta ROI | Group | 96.348 | 1 | 41 | <0.001 | <0.001 | 0.701 |
| Frontal-midline theta ROI | Condition | 4029.951 | 1 | 41 | <0.001 | <0.001 | 0.990 |
| Frontal-midline theta ROI | Interaction | 913.701 | 1 | 41 | <0.001 | <0.001 | 0.957 |
| Pz alpha power | Group | 322.259 | 1 | 41 | <0.001 | <0.001 | 0.887 |
| Pz alpha power | Condition | 644.100 | 1 | 41 | <0.001 | <0.001 | 0.940 |
| Pz alpha power | Interaction | 214.010 | 1 | 41 | <0.001 | <0.001 | 0.839 |

Note. Data are participant-level condition-summary measures unless otherwise specified. Mixed-design ANOVA used group as the between-subject factor and condition as the within-subject factor. Simple-effects P values were FDR-corrected using the Benjamini-Hochberg procedure where applicable. MDD = major depressive disorder; HC = healthy controls; ROI = region of interest.

## Supplementary Table S5b. Oscillatory FDR-corrected simple-effects results.

| Outcome | Comparison | n1 | n2 | t | P value | FDR-corrected P value | Cohen's d |
| --- | --- | --- | --- | --- | --- | --- | --- |
| Frontal-midline theta ROI | MDD vs HC within neutral | 22 | 21 | -2.796 | 0.010 | 0.010 | -0.837 |
| Frontal-midline theta ROI | MDD vs HC within incongruent | 22 | 21 | -18.893 | <0.001 | <0.001 | -5.703 |
| Frontal-midline theta ROI | incongruent vs neutral within MDD | 22 | 22 | 20.048 | <0.001 | <0.001 | 4.274 |
| Frontal-midline theta ROI | incongruent vs neutral within HC | 21 | 21 | 92.440 | <0.001 | <0.001 | 20.172 |
| Pz alpha power | MDD vs HC within neutral | 22 | 21 | 5.646 | <0.001 | <0.001 | 1.690 |
| Pz alpha power | MDD vs HC within incongruent | 22 | 21 | 23.602 | <0.001 | <0.001 | 7.113 |
| Pz alpha power | incongruent vs neutral within MDD | 22 | 22 | -5.841 | <0.001 | <0.001 | -1.245 |
| Pz alpha power | incongruent vs neutral within HC | 21 | 21 | -83.182 | <0.001 | <0.001 | -18.152 |

Note. Data are participant-level condition-summary measures unless otherwise specified. Mixed-design ANOVA used group as the between-subject factor and condition as the within-subject factor. Simple-effects P values were FDR-corrected using the Benjamini-Hochberg procedure where applicable. MDD = major depressive disorder; HC = healthy controls; ROI = region of interest.

## Supplementary Table S5c. Oscillatory descriptive statistics by group and condition.

| Outcome | Group | Condition | n | Mean | SD | SEM |
| --- | --- | --- | --- | --- | --- | --- |
| Frontal-midline theta ROI | MDD | neutral | 22 | 1.00 | 0.42 | 0.09 |
| Frontal-midline theta ROI | MDD | incongruent | 22 | 1.63 | 0.29 | 0.06 |
| Frontal-midline theta ROI | HC | neutral | 21 | 1.26 | 0.15 | 0.03 |
| Frontal-midline theta ROI | HC | incongruent | 21 | 3.02 | 0.18 | 0.04 |
| Pz alpha power | MDD | neutral | 22 | -0.54 | 0.30 | 0.06 |
| Pz alpha power | MDD | incongruent | 22 | -0.99 | 0.27 | 0.06 |
| Pz alpha power | HC | neutral | 21 | -0.92 | 0.10 | 0.02 |
| Pz alpha power | HC | incongruent | 21 | -2.56 | 0.15 | 0.03 |

Note. Descriptive statistics were computed from participant-level condition-summary values.

Large effect sizes should be interpreted in relation to the participant-level descriptive statistics provided in Supplementary Tables S5c and S6c.

## Supplementary Table S6a. Prefrontal ROI fNIRS mixed-design ANOVA results.

| Outcome | Effect | F | df1 | df2 | P value | FDR-corrected P value | Partial eta squared |
| --- | --- | --- | --- | --- | --- | --- | --- |
| HbO | Group | 571.120 | 1 | 41 | <0.001 | <0.001 | 0.933 |
| HbO | Condition | 1665.372 | 1 | 41 | <0.001 | <0.001 | 0.976 |
| HbO | Interaction | 642.753 | 1 | 41 | <0.001 | <0.001 | 0.940 |
| HbR | Group | 299.360 | 1 | 41 | <0.001 | <0.001 | 0.880 |
| HbR | Condition | 576.747 | 1 | 41 | <0.001 | <0.001 | 0.934 |
| HbR | Interaction | 282.312 | 1 | 41 | <0.001 | <0.001 | 0.873 |
| HbT | Group | 861.277 | 1 | 41 | <0.001 | <0.001 | 0.955 |
| HbT | Condition | 3603.077 | 1 | 41 | <0.001 | <0.001 | 0.989 |
| HbT | Interaction | 1183.484 | 1 | 41 | <0.001 | <0.001 | 0.967 |

Note. Data are participant-level condition-summary measures unless otherwise specified. Mixed-design ANOVA used group as the between-subject factor and condition as the within-subject factor. Simple-effects P values were FDR-corrected using the Benjamini-Hochberg procedure where applicable. MDD = major depressive disorder; HC = healthy controls; ROI = region of interest. HbO = oxygenated hemoglobin; HbR = deoxygenated hemoglobin; HbT = total hemoglobin.

## Supplementary Table S6b. Prefrontal ROI fNIRS FDR-corrected simple-effects results.

| Outcome | Comparison | n1 | n2 | t | P value | FDR-corrected P value | Cohen's d |
| --- | --- | --- | --- | --- | --- | --- | --- |
| HbO | MDD vs HC within neutral | 22 | 21 | -0.220 | 0.827 | 0.827 | -0.067 |
| HbO | MDD vs HC within incongruent | 22 | 21 | -25.047 | <0.001 | <0.001 | -7.748 |
| HbO | incongruent vs neutral within MDD | 22 | 22 | 19.815 | <0.001 | <0.001 | 4.225 |
| HbO | incongruent vs neutral within HC | 21 | 21 | 35.802 | <0.001 | <0.001 | 7.813 |
| HbR | MDD vs HC within neutral | 22 | 21 | -0.441 | 0.662 | 0.662 | -0.134 |
| HbR | MDD vs HC within incongruent | 22 | 21 | 17.442 | <0.001 | <0.001 | 5.412 |
| HbR | incongruent vs neutral within MDD | 22 | 22 | -10.803 | <0.001 | <0.001 | -2.303 |
| HbR | incongruent vs neutral within HC | 21 | 21 | -21.562 | <0.001 | <0.001 | -4.705 |
| HbT | MDD vs HC within neutral | 22 | 21 | -0.886 | 0.381 | 0.381 | -0.272 |
| HbT | MDD vs HC within incongruent | 22 | 21 | -33.004 | <0.001 | <0.001 | -10.166 |
| HbT | incongruent vs neutral within MDD | 22 | 22 | 27.470 | <0.001 | <0.001 | 5.857 |
| HbT | incongruent vs neutral within HC | 21 | 21 | 53.402 | <0.001 | <0.001 | 11.653 |

Note. Data are participant-level condition-summary measures unless otherwise specified. Mixed-design ANOVA used group as the between-subject factor and condition as the within-subject factor. Simple-effects P values were FDR-corrected using the Benjamini-Hochberg procedure where applicable. MDD = major depressive disorder; HC = healthy controls; ROI = region of interest. HbO = oxygenated hemoglobin; HbR = deoxygenated hemoglobin; HbT = total hemoglobin.

## Supplementary Table S6c. Prefrontal ROI fNIRS descriptive statistics by group and condition.

| Outcome | Group | Condition | n | Mean | SD | SEM |
| --- | --- | --- | --- | --- | --- | --- |
| HbO | MDD | neutral | 22 | 0.05 | 0.01 | 0.00 |
| HbO | MDD | incongruent | 22 | 0.15 | 0.03 | 0.01 |
| HbO | HC | neutral | 21 | 0.05 | 0.01 | 0.00 |
| HbO | HC | incongruent | 21 | 0.47 | 0.05 | 0.01 |
| HbR | MDD | neutral | 22 | -0.02 | 0.01 | 0.00 |
| HbR | MDD | incongruent | 22 | -0.05 | 0.01 | 0.00 |
| HbR | HC | neutral | 21 | -0.02 | 0.01 | 0.00 |
| HbR | HC | incongruent | 21 | -0.19 | 0.03 | 0.01 |
| HbT | MDD | neutral | 22 | 0.03 | 0.01 | 0.00 |
| HbT | MDD | incongruent | 22 | 0.10 | 0.01 | 0.00 |
| HbT | HC | neutral | 21 | 0.03 | 0.01 | 0.00 |
| HbT | HC | incongruent | 21 | 0.28 | 0.02 | 0.00 |

Note. Descriptive statistics were computed from participant-level condition-summary values.

Large effect sizes should be interpreted in relation to the participant-level descriptive statistics provided in Supplementary Tables S5c and S6c.

## Supplementary Table S7. Clinical correlation and sensitivity-analysis results.

| Analysis | Variable 1 | Variable 2 | Sample | n | r | 95% CI | P value / Alpha | FDR-corrected P value / Power | Interpretation / Minimum detectable effect |
| --- | --- | --- | --- | --- | --- | --- | --- | --- | --- |
| Part A. Clinical correlation analysis |  |  |  |  |  |  |  |  |  |
| Pooled | P300 amplitude | HAMD | All participants | 43 | -0.461 | -0.669 to -0.186 | 0.002 | 0.004 | Exploratory pooled association; interpret cautiously because diagnostic groups differed in HAMD. |
| Within-MDD | P300 amplitude | HAMD | MDD | 22 | 0.289 | -0.151 to 0.633 | 0.192 | 0.192 | Within-MDD association was not significant. |
| Part B. Sensitivity analysis |  |  |  |  |  |  |  |  |  |
| Independent-samples comparison | MDD n = 22, HC n = 21 |  |  |  |  |  | alpha = 0.05 | power = 0.80 | Minimum detectable Cohen's d = 0.88 |
| Pooled correlation | n = 43 |  |  |  |  |  | alpha = 0.05 | power = 0.80 | Minimum detectable r = 0.41 |
| Within-MDD correlation | n = 22 |  |  |  |  |  | alpha = 0.05 | power = 0.80 | Minimum detectable r = 0.55 |

Note. Data are participant-level condition-summary measures unless otherwise specified. Mixed-design ANOVA used group as the between-subject factor and condition as the within-subject factor. Simple-effects P values were FDR-corrected using the Benjamini-Hochberg procedure where applicable. MDD = major depressive disorder; HC = healthy controls; ROI = region of interest. HbO = oxygenated hemoglobin; HbR = deoxygenated hemoglobin; HbT = total hemoglobin.
